# *Streptococcus suis* contributes to inguinal lymph node lesions in piglets after highly pathogenic porcine reproductive and respiratory syndrome virus infection

**DOI:** 10.3389/fmicb.2023.1159590

**Published:** 2023-04-27

**Authors:** Shujie Wang, Min Xu, Kongbin Yang, Ying Zhang, Siqi Li, Yan-Dong Tang, Jinliang Wang, Chaoliang Leng, Tongqing An, Xuehui Cai

**Affiliations:** ^1^State Key Laboratory for Animal Disease Control and Prevention, Harbin Veterinary Research Institute, Chinese Academy of Agricultural Sciences, Harbin, China; ^2^Heilongjiang Provincial Key Laboratory of Veterinary Immunology, Harbin, China; ^3^Sinopharm Animal Health Corporation Ltd., Wuhan, China; ^4^Neurosurgery Department, The Fifth Affiliated Hospital of Guangzhou Medical University, Guangzhou, China; ^5^Shandong Binzhou Animal Science and Veterinary Medicine Academy, Binzhou, China; ^6^Henan Key Laboratory of Insect Biology in Funiu Mountain, Henan Provincial Engineering Laboratory of Insects Bio-Reactor, China-UK-NYNU-RRes Joint Laboratory of Insect Biology, Nanyang Normal University, Nanyang, China

**Keywords:** HP-PRRSV, *Streptococcus suis*, inguinal lymph nodes, apoptosis, pyroptosis

## Abstract

The swine pathogens porcine reproductive and respiratory syndrome virus (PRRSV) and *Streptococcus suis* have both been reported to cause damage to the immune organs. Inguinal lymph node (ILN) injury has been reported in PRRSV-infected pigs with secondary *S. suis* infection, but not much is known about the mechanism. In this study, secondary *S. suis* infection after highly pathogenic (HP)-PRRSV infection caused more severe clinical symptoms, mortality, and ILN lesions. Histopathological lesions were seen in ILNs with a marked decrease in lymphocyte numbers. Terminal deoxynucleotidyl transferase (TdT)-mediated de-oxyuridine triphosphate (dUTP)-biotin nick end-labeling (TUNEL) assays revealed that HP-PRRSV strain HuN4 alone induced ILN apoptosis, but dual-infection with *S. suis* strain BM0806 induced greater levels of apoptosis. Besides, we found that some HP-PRRSV-infected cells underwent apoptosis. Furthermore, anti-caspase-3 antibody staining confirmed that ILN apoptosis was mainly induced by a caspase-dependent pathway. Pyroptosis was also observed in HP-PRRSV-infected cells, and there was more pyroptosis in piglets infected with HP-PRRSV alone compared with those with secondary *S. suis* infection, and HP-PRRSV-infected cells underwent pyroptosis. Altogether, this is the first report to identify pyroptosis in ILNs and which signaling pathway is related to ILN apoptosis in single or dual-infected piglets. These results contribute to a better understanding of the pathogenic mechanisms during secondary *S. suis* infection.

## Introduction

Porcine reproductive and respiratory syndrome virus (PRRSV) is a positive-sense, single-stranded, enveloped RNA virus (Firth et al., 2011; Wang et al., 2016) belonging to the family *Arteriviridae* and genus *Betaarterivirus* (Dokland, 2010; Ma et al., 2018). PRRSV was first discovered in the United States in 1987 (Rossow, 1998), whereas in China, classic PRRSV and highly pathogenic (HP)-PRRSV strains were isolated in 1996 and 2006 (Li et al., 2007; Jiang et al., 2020), respectively. Classic PRRSV can cause acute respiratory disease in piglets and fever, breathing difficulties and miscarriage in sows (Van Reeth, 1997; Johnson et al., 2004). HP-PRRSV is different from the classic strain, causing severe disease not only in piglets but also in adult pigs, with strong pathogenicity and high mortality resulting in huge economic losses for affected pig farms (Hu et al., 2013; Do et al., 2015; Chen et al., 2016).

Many studies have demonstrated the immunosuppressive nature of PRRSV (Fan et al., 2015; Zhou et al., 2021). PRRSV infection can cause atrophy of the thymus, in which a large number of CD3^+^ T cells undergo apoptosis, decreasing the number of CD4^+^ and CD8^+^ T cells in peripheral blood (Li et al., 2014; Wang et al., 2015). PRRSV has a strong affinity for alveolar macrophages and can infect and replicate within them (Sun et al., 2022). Part of circulating antibodies can enhance viral infection and replication in macrophages (Shi et al., 2018, 2019). Meanwhile, PRRSV destroys macrophage function and inhibits their non-specific bactericidal activities (Jung et al., 2009), which can lead to serious secondary bacterial infections.

Inguinal lymph nodes (ILNs) are peripheral immune organs that defend against foreign bodies, producing antibodies and participating in the systemic immune response to pathogens (Sever et al., 2012). Immunosuppressive diseases are characterized by the destruction of immune organs (Wang et al., 2021), and PRRSV is known to damage immune organs including the thymus, bone marrow, spleen, and ILNs (Feng et al., 2001; Wang G. et al., 2020; Wang et al., 2022). Moreover, *Streptococcus suis* has also been reported to damage the thymus of pigs in recent years (Wang S. et al., 2020). However, reports of the mechanism for HP-PRRSV damage to peripheral immune organs such as ILNs, and specifically the involvement of secondary *S. suis* infection in ILN injury are less reported (Feng et al., 2001; Wang et al., 2014).

The purpose of this study was to explore the mechanism of ILN pathogenesis, both by HP-PRRSV alone or with secondary *S. suis* infection, and explore the differences in ILN apoptosis/pyroptosis in infected piglets.

## Materials and methods

### Ethics statement

All animal experiments were approved by the Committee on the Ethics of Animal Experiments of the Harbin Veterinary Research Institute of the Chinese Academy of Agricultural Sciences (CAAS), China. Piglet dual-infection experiments (approval number 200805-06) were carried out in the animal biosafety level 2 facilities at the Harbin Veterinary Research Institute, CAAS.

### Bacterial and viral strains

The HP-PRRSV HuN4 strain (Wang et al., 2015) and *S. suis* serotype 7 strain BM0806 (Wang S. et al., 2020) used in the current study were stored in our lab. PRRSV HuN4 was passed three times on MARC-145 cells in Dulbecco’s modified Eagle’s medium (DMEM) with 8% fetal calf serum (Excel Bio, Shanghai, China) at 37°C. *Streptococcus suis* BM0806 was grown on sheep blood agar plates at 37°C for 20 h, and three colonies were inoculated into Todd-Hewitt broth (THB; HAIBO, Qingdao, China) and incubated for 12 h at 37°C with agitation.

### Animal experiments

**Figure 1:**
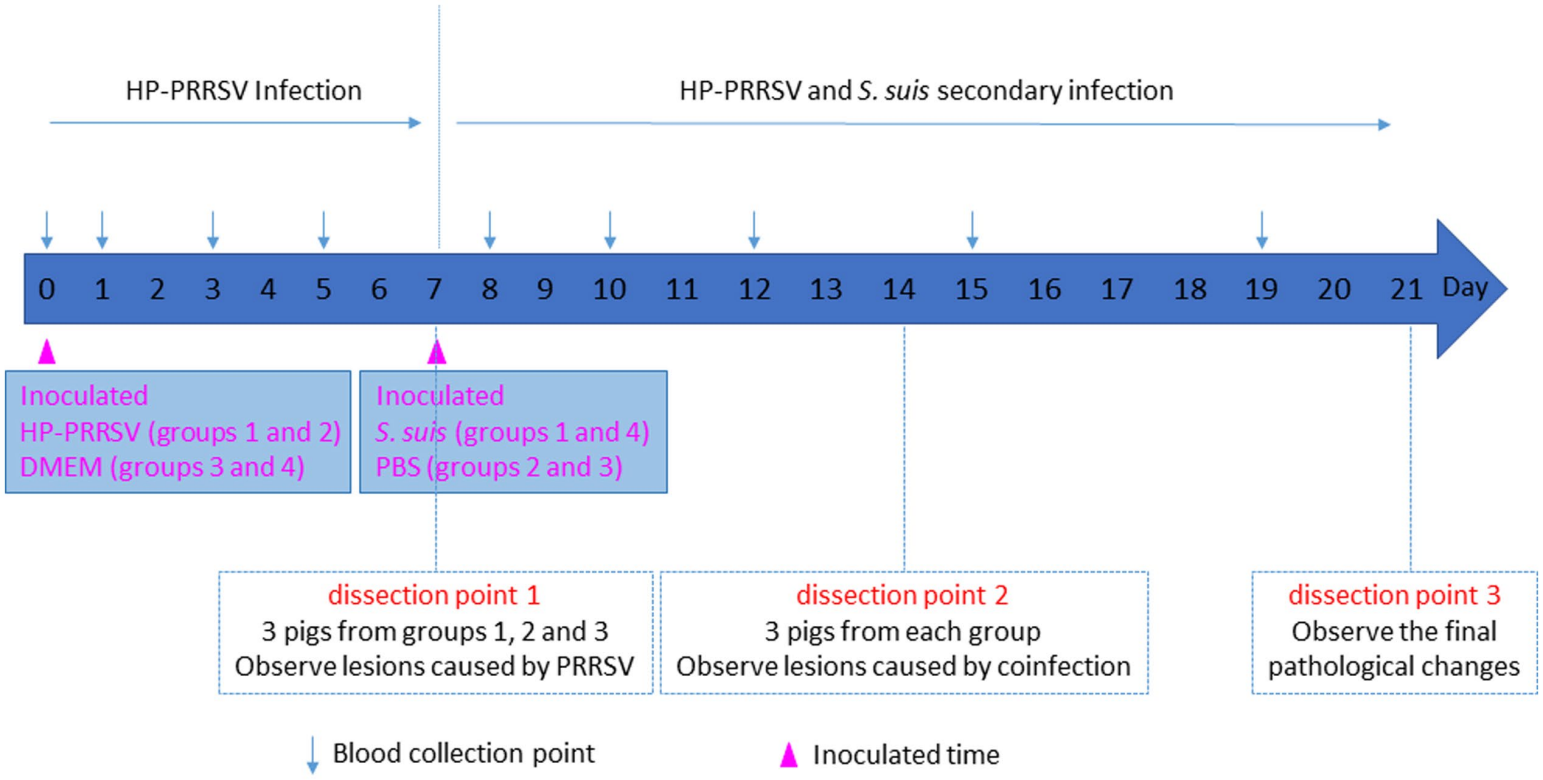
Schematic diagram of piglet infection experiment.

A total of 40, 21-day-old PRRSV- and *S. suis*-negative crossbred piglets were randomly divided into four groups and housed separately in isolation rooms. As shown in Figure 1, after 1 week (day 0), groups 1 (*n* = 12 piglets) and 2 (*n* = 10 piglets) were inoculated with 3 mL HP-PRRSV HuN4 (2 × 10^5^ TCID_50_ total, 2 mL intranasal and 1 mL intramuscular inoculation); groups 3 and 4 (*n* = 9 piglets each) were inoculated with 3 mL DMEM. On day 7, groups 1 and 4 were inoculated with 3 mL *S. suis* BM0806 (3 × 10^9^ colony-forming units total, 2 mL intranasal and 1 mL intramuscular inoculation). Pigs were monitored daily for clinical signs including fever, lethargy, sneezing, joint swelling, lameness, central nervous system disease, and changes of skin. Three piglets from each group were humanely euthanized on day 7, 14, and 21. Lung and ILN samples were collected and macroscopic lesions were scored on a scale from 0 to 3 using the system outlined in Table 1. During the experiment, any piglets exhibiting extreme lethargy were euthanized humanely.

**Table 1:** Macroscopic pathohistological score.

Parameter	Score 0	Score 1	Score 2	Score 3
Lung	No visible lesions	Slight interstitial pneumonia or congestion	Interstitial pneumonia with local emphysema/consolidation	Interstitial pneumonia with emphysema/consolidation/severe bleeding
ILN	No visible lesions	Slight enlargement	Swelling and hemorrhage	Swelling and severe hemorrhage

### Histopathology examinations

Inguinal lymph node samples from sacrificed experimental animals were placed in 4% formalin in phosphate buffer and fixed for 7 days, embedded in paraffin and cut into 8-μm-thick slices prior to staining with hematoxylin and eosin (H&E). Tissue sections were observed by a professional pathologist on an inverted light microscope (Carl Zeiss, Munich, Germany). For morphometric analysis, the average number of lymphocytes in H&E sections was determined from counts of 10 microscope fields (400×). The mean number of lymphocytes per mm^2^ was calculated.

### Confocal microscopy

Inguinal lymph node samples from each group were collected and sectioned in 8-μm-thick slices for immunofluorescence. An *In situ* Cell Death Detection Kit (Roche, Mannheim, Germany) was used to detect apoptosis in ILNs by terminal deoxynucleotidyl transferase (TdT)-mediated deoxyuridine triphosphate (dUTP)-biotin nick end-labeling (TUNEL) assay. To identify the apoptosis signaling pathway, rabbit anti-mouse caspase-3 antibody (1:500, Cell Signaling, Danvers, United States), rabbit anti-mouse apoptosis-inducing factor (AIF) monoclonal antibody (1:50, Abcam, Cambridge, United Kingdom), and Alexa Fluor 568-conjugated goat anti-rabbit antibody (1:500, Sigma, Saint Louis, United States) were used. Rabbit polyclonal antiserum against *S. suis* serotype 7 (1:50, SSI, Denmark), FITC-conjugated goat anti-rabbit antibody (1:500, Sigma), monoclonal antibody (mAb) against HP-PRRSV N protein (1:500, produced in lab), and Alexa Fluor 488 rabbit anti-mouse antibody (1:1,000, Sigma) were used. To identify pyroptosis, rabbit anti-mouse gasdermin-D-N/C (GSDMD-N/C; 1:50, Abcam), mouse polyclonal antiserum against *S. suis* serotype 7 (1:50, made in lab) and Alexa Fluor 568/488-conjugated goat anti-rabbit/mouse antibody (1:500, Sigma) were used. Nuclei were stained with 4-6-diamidino-2-phenylindole (DAPI, Sigma), and sections were observed by confocal laser scanning microscopy with fast airyscan LSM880 (Carl ZEISS, Jena, Germany). For morphometric analysis, the apoptotic or pyroptotic cells in 8 microscopy fields of the ILN sections were counted. The mean number of labeled cells per mm^2^ was calculated.

### Statistical analysis

Data were expressed as the mean ± standard deviation and analyzed by GraphPad Prism software (version 5.01; GraphPad Software Inc.). Differences between groups were assessed by one-way ANOVA and Tukey’s multiple-comparison test or Fisher’s exact test for categorical variables. A *p* value less than 0.05 was considered statistically significant.

## Results

### Dual-infection with *Streptococcus suis* caused more severe clinical symptoms and mortality in PRRSV-infected piglets

**Figure 2:**
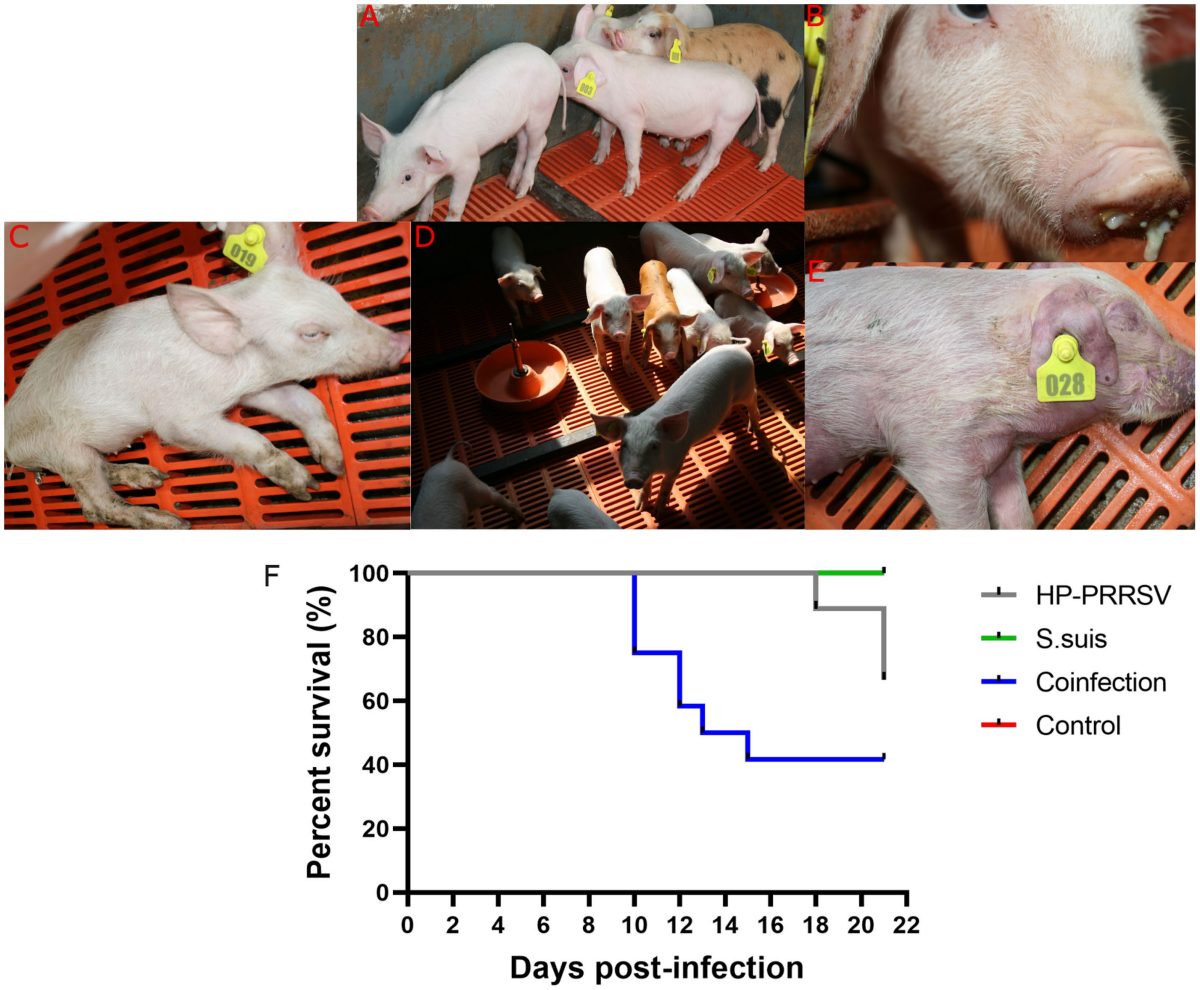
Clinical signs and mortality percent in piglets during the animal infection experiment. Clinical signs observed during experiment in **(A)** mock-infected control group; **(B)** Nasal discharge; **(C)** Anorexia, depression, and emaciation were seen in HP-PRRSV-infected piglets; **(D)** mild cough was seen in *Streptococcus suis*-infected group; **(E)** cyanotic limbs and ears were seen in co-infected group; **(F)** Survival percent in different experimental groups.

To investigate whether dual-infection with *S. suis* caused more severe clinical symptoms and mortality in PRRSV-infected piglets, we used *S. suis* BM0806 to infect PRRSV-infected piglets. As shown in Figure 2, piglets in the mock-infected control group did not show any clinical signs during the experiment. In the PRRSV HuN4-infected group, piglets developed symptoms of sneezing, cough, and diarrhea by 6 days post-infection (dpi), and anorexia, lying down, weight loss, wheezing, and muscle tremors at 11 dpi, one piglet died at 18, 21, and 22 dpi, respectively; mortality was 33%. In the group infected with *S. suis* BM0806 alone, several pigs (5/10) showed mild symptoms of cough and sneezing during first 3 days after infection. In the dual-infected group, piglets developed symptoms of anorexia, dyspnea, emaciation, tremors, and unsteadiness at 9 dpi, and three piglets died at 10 dpi, two piglets died at 12 dpi, one piglet died at 13 dpi with cyanotic limbs and ears, and one piglet exhibiting extreme lethargy was euthanized humanely on 15 dpi; overall mortality was 58.3%.

**Figure 3:**
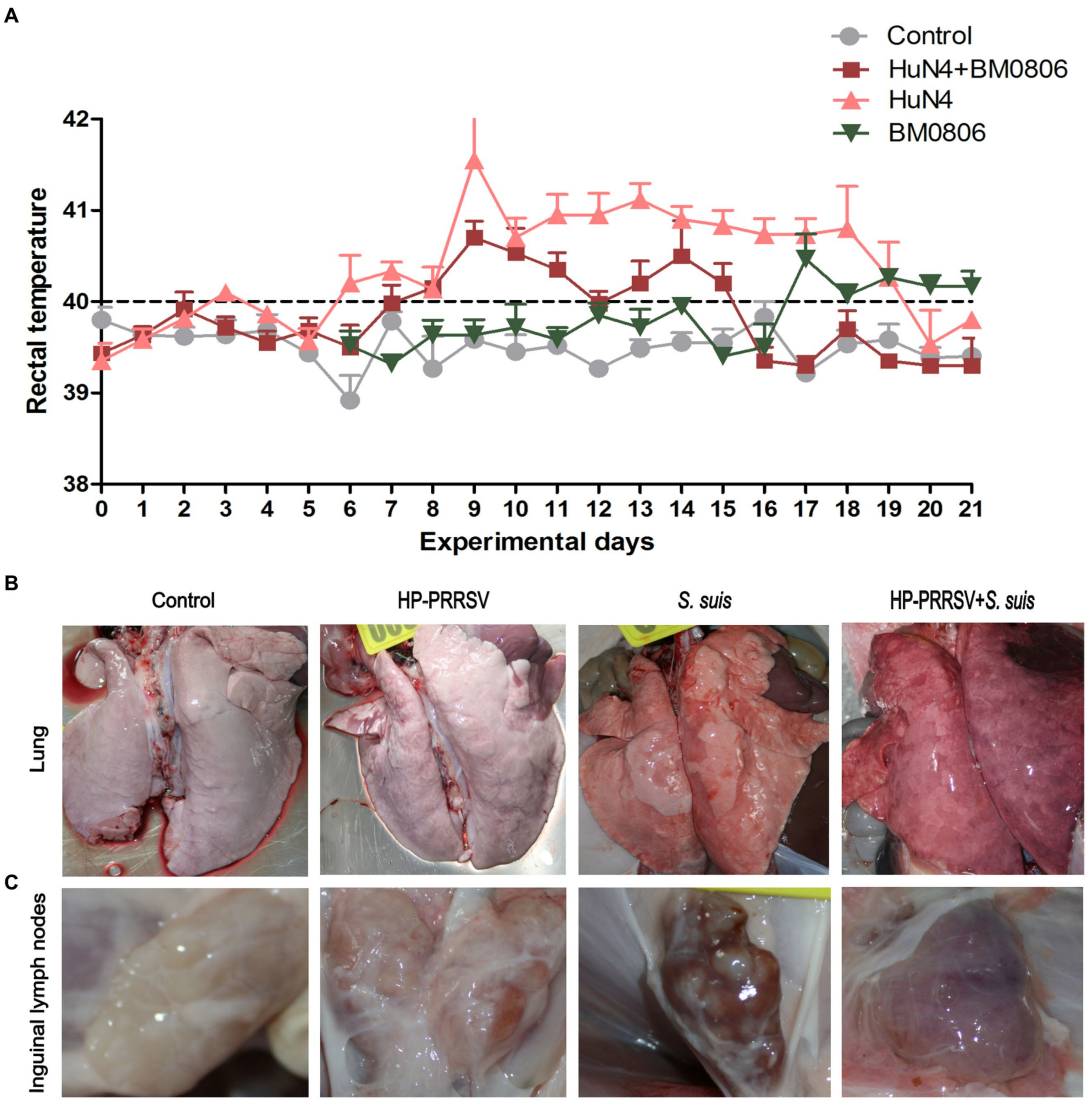
Gross lesions in lung and inguinal lymph nodes after infection with HP-PRRSV and/or *Streptococcus suis*. **(A)** Daily average rectal temperature of piglets from each treatment group. The mean ± S.D. is plotted from all piglets on each experimental day. Piglets were mock infected (Control) or infected with HP-PRRSV and/or *S. suis*. Representative gross pathological lesions are depicted from **(B)** the lungs and **(C)** the inguinal lymph nodes of each experimental group.

The rectal temperature of piglets in the PRRSV HuN4-infected group spiked (> 41.5°C) on day 9, remained above 40.7°C from day 10 to 18, and fell below 40°C on days 20 and 21 (Figure 3A). The rectal temperature of piglets in the dual-infected group rose above 40.2°C from day 8 to 15 (except on day 12), and reached the peak (40.7°C) on day 9. The temperature dropped below 40°C and remained stable from day 16 to 21. In the group infected with *S. suis* BM0806 alone, the rectal temperature of piglets was above 40.0°C from day 17 to 21. No significant increase in rectal temperature was observed in the control group.

### More severe gross and histopathologic lesions in ILNs of dual-infected piglets

The macroscopic pathohistological score for lungs and ILNs in each experimental groups are presented in Table 2. The main visible organ lesions in PRRSV HuN4-infected piglets at 14 dpi were clearly found in lung (Figure 3B) and ILNs (Figure 3C). Piglets developed marked lobular interstitial pneumonia with fleshy consolidation in the sharp lobes of the lung. Gross lesions of the ILNs included swelling and hemorrhage. The main organ lesions found in the dual-infected piglets that died at 12 and 13 dpi were interstitial pneumonia with severe bleeding in the lungs and adhesions to the body cavity, severe bacterial infection and massive purulent fluid in the abdominal cavity, with swelling and hemorrhage in the ILNs. In the piglets infected with *S. suis* BM0806 alone, those sacrificed at 14 dpi had interstitial pneumonia or congestion with local emphysema in both lungs, hemorrhage and meningeal hemorrhage in the ILNs.

**Table 2:** Macroscopic pathohistological score for lungs and ILNs.

Goups no. (*n*)	Designation	Lungs	ILNs
1 (12)	Co-infection	2.5^A^	2.5^A^
2 (10)	HP-PRRSV	2.2^B^	2.0^B^
3 (9)	PBS	0^B^	0^B^
4 (9)	*S. suis*	1.5^C^	2.1^B^

^A–C^Different superscripts within a column indicate significant differences (*p* < 0.05) determined by Fisher’s exact test.

**Figure 4:**
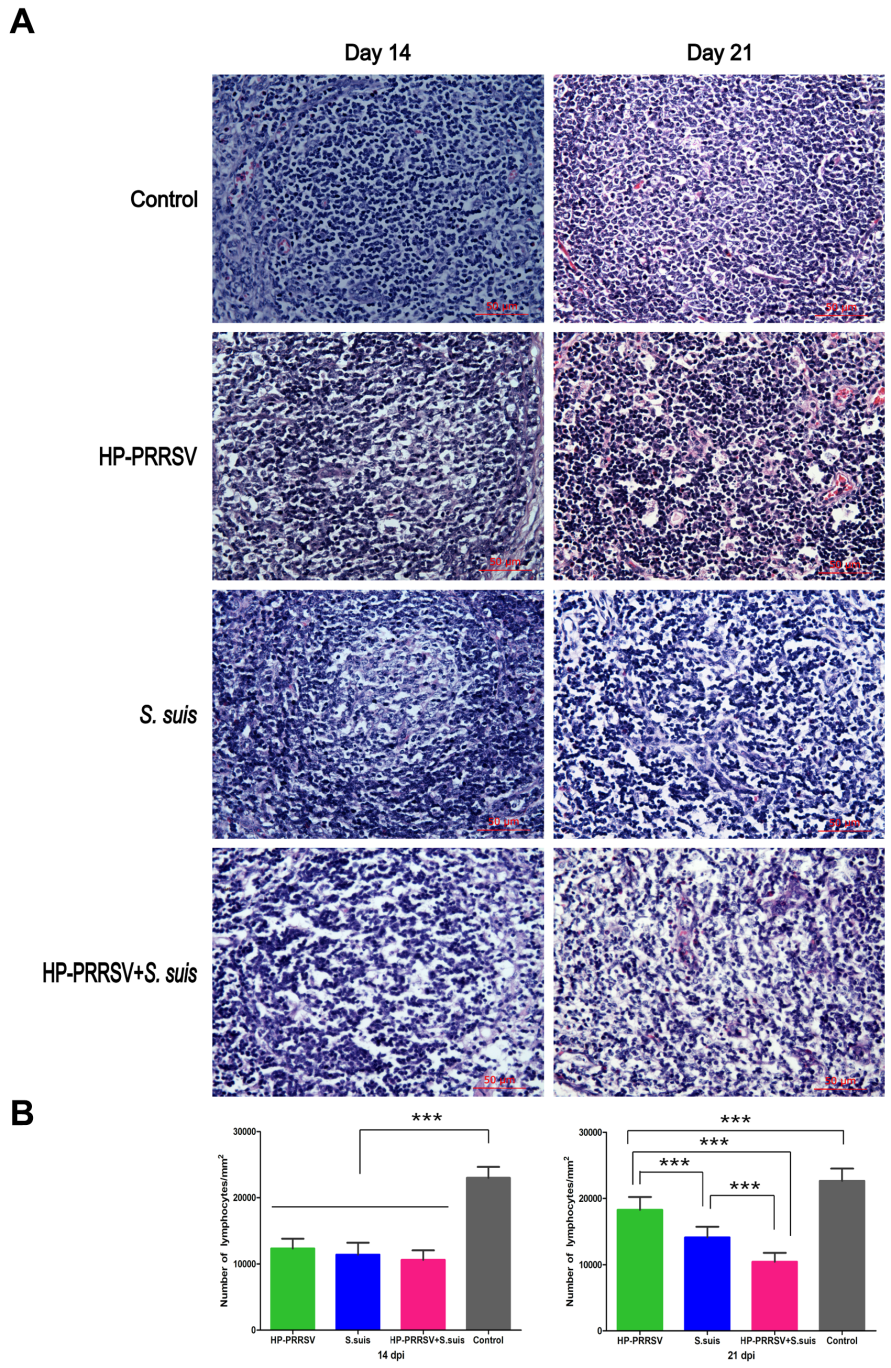
Histopathological lesions induced in the inguinal lymph nodes of infected piglets. **(A)** Representative hematoxylin & eosin staining in the inguinal lymph nodes of piglets mock infected (Control) or infected with HP-PRRSV and/or *Streptococcus suis.* **(B)** Number of lymphocytes in ILNs from different groups of pigs on 14 and 21 dpi. On 14 dpi, the number of lymphocytes decreased significantly in the inguinal lymph nodes from three infected groups compared with control group. On 21 dpi, the number of lymphocytes in the single-infected groups recovered, with no significant recovery in dual-infected group. ^***^p < 0.001.

Representative histopathology for each treatment group is shown in Figure 4A. The main microscopic lesions in ILNs from PRRSV HuN4-infected piglets were a marked reduction in number of lymphocytes and visibly small vacuoles. In dual-infected piglets, the main ILN lesions were massive disintegration and necrosis of lymphocytes, macrophage infiltration and intense plasma cell proliferation. In piglets infected with *S. suis* BM0806 alone, the main ILN lesions were lymphocyte depletion and necrosis, lymph node nodules that were not obvious, and some eosinophilic infiltration into the paracortical areas. Compared with the control group, the lymphocytes in all three infected groups were significantly reduced on 14 dpi, but there was no statistical difference among them on 14 dpi (Figure 4B). On 21 dpi, the number of lymphocytes in the ILNs in single-infected pigs recovered, but there was no recovery in the dual-infected group, compared with the number of lymphocytes in the ILNs of the pigs on 14 dpi.

### More apoptosis in ILNs of dual-infected piglet than PRRSV-infected piglets

**Figure 5:**
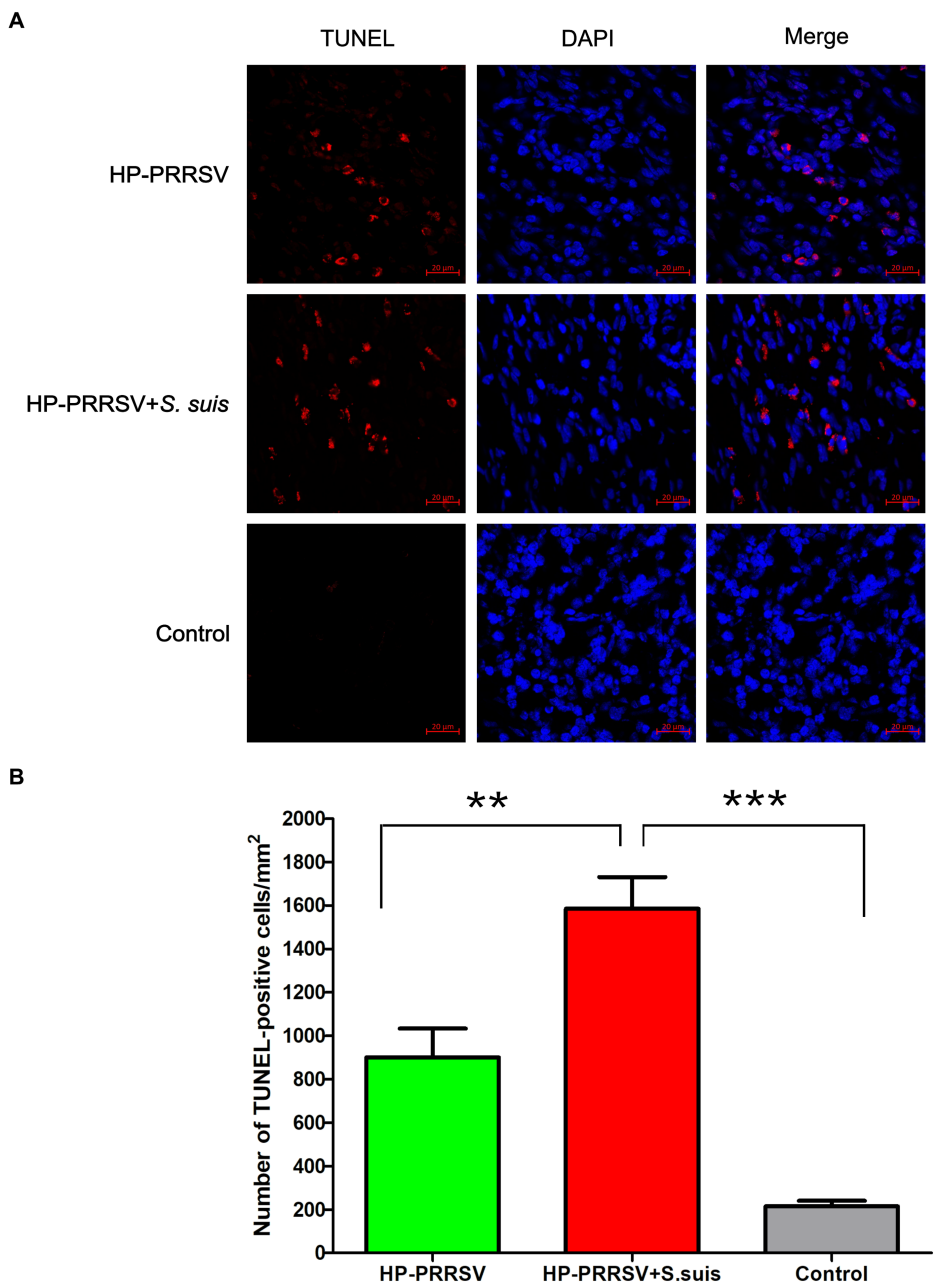
Identification of apoptotic cells in the inguinal lymph nodes of infected pigs. Sections of inguinal lymph node were prepared from piglets mock infected (Control) or infected by HP-PRRSV with or without *Streptococcus suis* dual-infection. **(A)** Apoptotic cells (TUNEL) were detected with the *In situ* Cell Death Detection Kit, and cell nuclei were stained with DAPI. **(B)** Number of apoptotic cells in ILNs from different groups of pigs. ^**^p < 0.01, ^***^p < 0.001.

In the following experiments, in order to explore the role of bacteria in ILN injury, we mainly compared the PRRSV single-infection group with the dual-infected. To investigate the cause of the massive decrease in the number of lymphocytes in the ILNs of infected piglets, apoptosis was detected using TUNEL assays. TUNEL-positive signals were observed in ILN sections from infected pigs (Figure 5A), and a large number of ILN cells underwent apoptosis in both the PRRSV-infected (*p* < 0.01) and dual-infected (*p* < 0.001) piglets compared with the mock-infected controls (Figure 5B). Moreover, the number of apoptotic cells in the dual-infected group was higher than in the group infected with PRRSV alone (*p* < 0.01).

### Colocalization of PRRSV HuN4-infected cells and apoptotic cells

**Figure 6:**
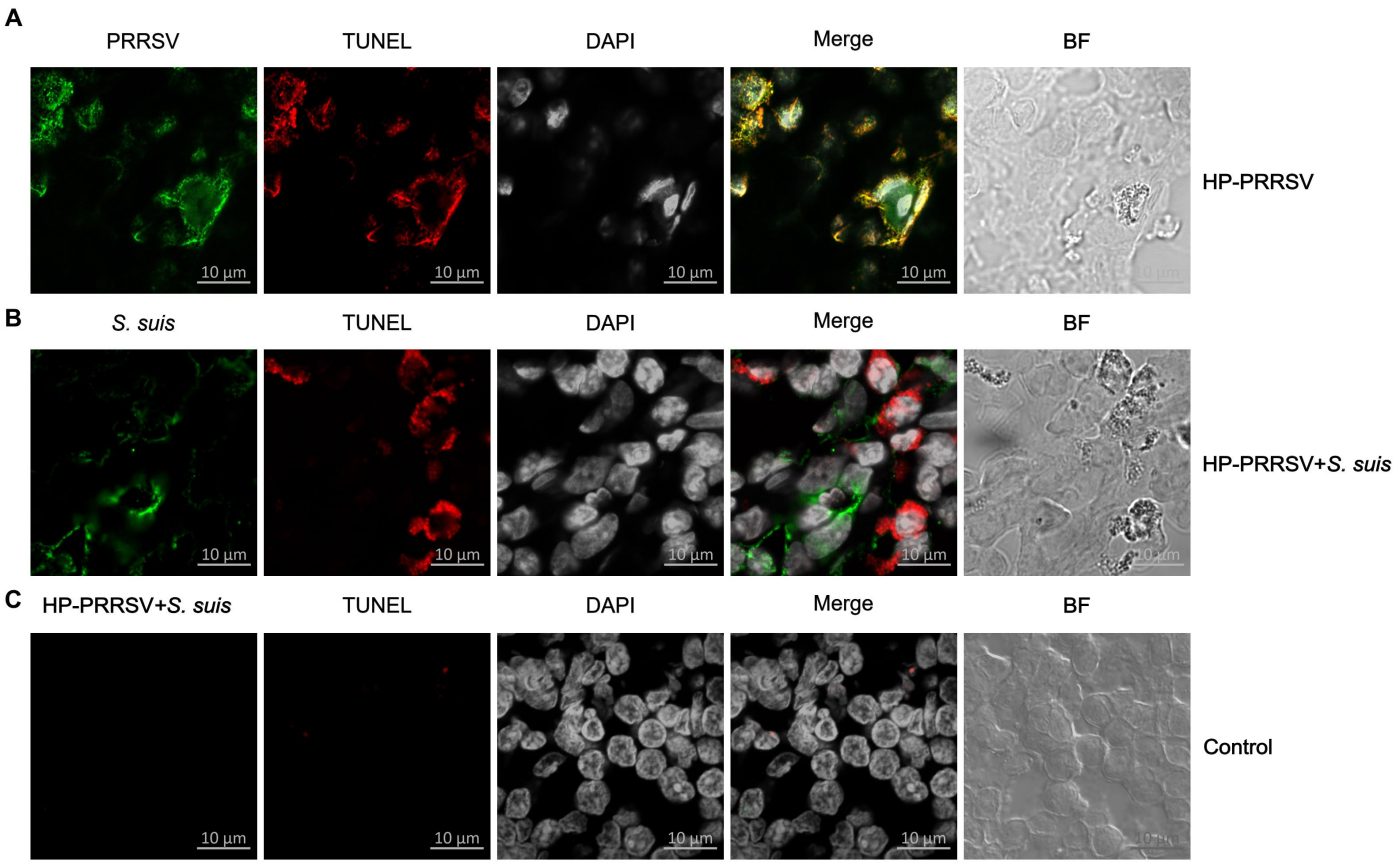
Colocalization of HP-PRRSV and *Streptococcus suis* in apoptotic inguinal lymph node cells. Sections of inguinal lymph node were prepared from piglets mock infected (Control) or infected by HP-PRRSV with or without *S. suis* dual-infection. Sections were incubated with antibodies against **(A)** HP-PRRSV N protein or **(B)** *S. suis* or **(C)** HP-PRRSV + *S. suis* and corresponding secondary antibodies. Apoptotic cells (TUNEL) were detected with the *In Situ* Cell Death Detection Kit, and cell nuclei were stained with DAPI. Bright field images are also included (BF).

Double immunofluorescence staining was used to determine whether HP-PRRSV or *S. suis* signals colocalized with apoptotic cells. ILN sections were incubated with HP-PRRSV or *S. suis* polyclonal antibodies, and apoptosis was detected using a TUNEL assay. HP-PRRSV-infected cells colocalized with apoptotic cells, indicating that HP-PRRSV-infected cells underwent apoptosis (Figure 6A). On the other hand, *S. suis-* and TUNEL-positive signal was not observed in the same cells, indicating a lack of apoptosis in *S. suis*-adhered cells (Figure 6B). Figure 6C is the ILN from control pig.

### PRRSV HuN4 and *Streptococcus suis* BM0806 mainly induce caspase-dependent apoptosis in ILNs

**Figure 7:**
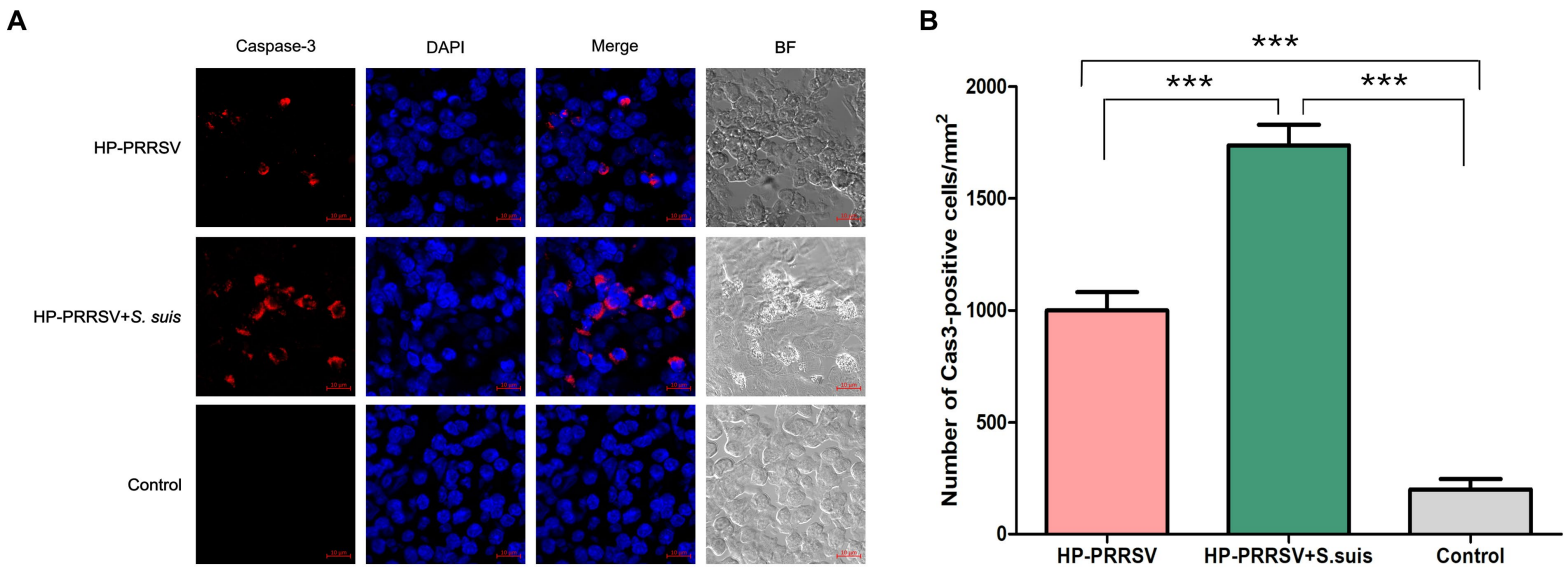
Porcine reproductive and respiratory syndrome virus (PRRSV) and *Streptococcus suis* induce caspase-dependent apoptosis in the inguinal lymph nodes of dual-infected piglets. Sections of inguinal lymph node were prepared from piglets mock infected (Control) or infected by HP-PRRSV with or without *S. suis* dual-infection. Cells were stained with **(A)** rabbit anti-mouse caspase-3 antibody and Alexa Fluor 568-conjugated goat anti-rabbit antibody. Nuclei were stained by DAPI; bright field images are also included (BF). **(B)** Number of caspase 3-positive cells in ILNs from different experimental groups. ^***^p < 0.001, compared with control.

Caspase-3 is considered a biochemical hallmark of apoptosis (Wang S. et al., 2020). We found caspase-3 expression in apoptotic cells in ILNs from piglets either infected with PRRSV alone, or dual-infected with *S. suis* (Figure 7A). Consistent with the TUNEL results, the number of apoptotic cells in the dual-infected group was greater than in the group infected with PRRSV alone (Figure 7B). Apoptosis-inducing factor (AIF) mainly mediates the p53 apoptosis pathway (Ding et al., 2018). We attempted to detect AIF protein expression in the infected groups, and no obvious difference of fluorescent signals was detected in ILNs of any of the infected piglets relative to mock-infected controls (data not shown). These results suggest that HP-PRRSV HuN4 induce caspase-dependent apoptosis in ILNs from infected piglets.

### More pyroptosis signal was observed in ILNs of piglets infected by PRRSV alone

**Figure 8:**
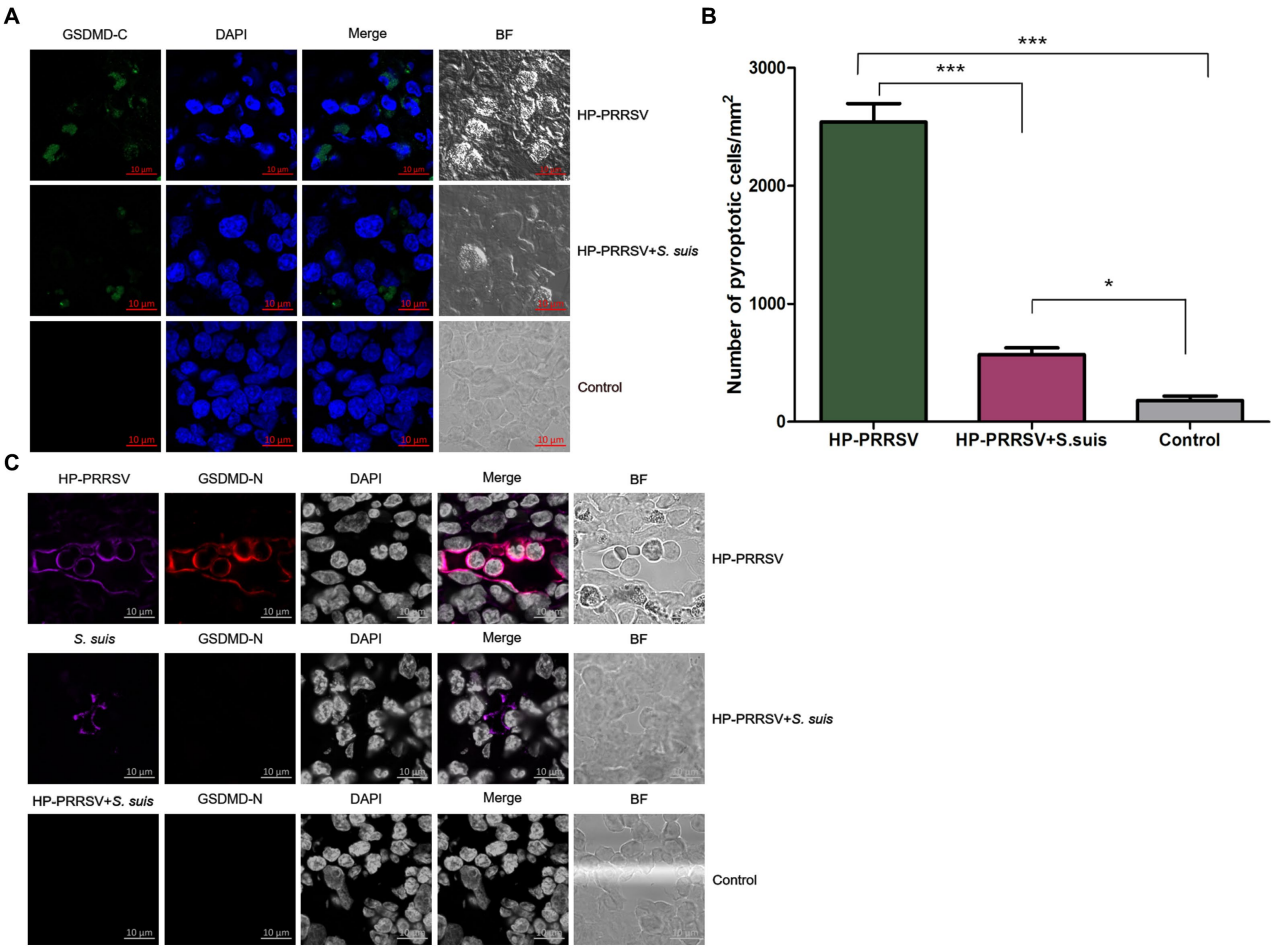
Characterization of pyroptosis in the inguinal lymph nodes of infected piglets. Sections of inguinal lymph node were prepared from piglets mock infected (Control) or infected by HP-PRRSV with or without *Streptococcus suis* dual-infection. Cells were stained with **(A)** rabbit anti-mouse GSDMD-C (green) antibody. **(B)** Number of pyroptotic cells in ILNs from different groups of pigs. **(C)** Cells were stained for mouse HP-PRRSV (purple) N protein mAb or *S. suis* (purple) mouse antibody and rabbit anti-mouse GSDMD-N (red). Nuclei were stained by DAPI; bright field images are also included (BF). ^*^p < 0.05, ^***^p < 0.001.

Pyroptosis is a form of programmed necrosis that is mediated by gasdermin D (GSDMD; Shi et al., 2017). To investigate whether pyroptosis occurred in the ILNs of HP-PRRSV-infected or PRRSV/*S. suis* dual-infected piglets, we used confocal microscopy to detect the GSDMD C-terminal domain in tissue sections. In contrast to mock-infected piglets, both PRRSV-infected and dual-infected piglets displayed ILN pyroptosis (Figure 8A), with much greater signal in those infected by PRRSV alone (Figure 8B).

To determine whether infected cells were undergoing pyroptosis, double immunofluorescence staining experiments were carried out using specific anti-PRRSV or anti-*S. suis* antibodies along with anti-GSDMD-N-terminal domain antibody. Some HP-PRRSV-infected cells colocalized with pyroptotic cells, whereas *S. suis*-adhered cells from ILNs of dual-infected piglets did not show any GSDMD-N signal (Figure 8C). These findings suggest that HP-PRRSV-infected cells may undergo pyroptosis.

## Discussion

Porcine reproductive and respiratory syndrome virus is the most economically important pathogen in pigs worldwide (Lunney et al., 2016), and it has developed multiple mechanisms to evade the host immune response (Costers et al., 2008; Sang et al., 2011). After infecting alveolar macrophages in the lungs, PRRSV spreads to the macrophages present in tissues, resulting in destruction of lymphocytes and mucosal barriers. The synergistic pathogenesis of PRRSV and *S. suis* has been reported (Galina et al., 1994; Thanawongnuwech et al., 2000; Sun et al., 2020). In our study, the dual-infected group had a higher mortality rate (58.3%) than the PRRSV-infected group (33%), which is consistent with previous reports (Feng et al., 2001; Li et al., 2019). It could be that the ability of infected macrophages to engulf bacteria is compromised after the viral infection. Our observed mortality following infection with *S. suis* (58.3%) was comparable to that reported by Feng et al. (2001) (91%), who infected sows late in pregnancy to have PRRSV-infected piglets at birth, with bacterial challenge at 5 days-of-age. Any difference in mortality may stem from variation in piglet age and route of PRRSV or *S. suis* infection.

The results of the current study are similar to previous findings that piglets infected with HP-PRRSV or PRRSV develop ILN lesions (Rossow et al., 1995; Feng et al., 2001; Wang et al., 2014). Furthermore, we showed the impact of secondary *S. suis* infection on the ILN lesions induced by HP-PRRSV. In the acute infection period, the amount of lymphocyte depletion in all infected groups was very serious, however, after which the amount of lymphocyte in the single infection group including HP-PRRSV and *S. suis* infection alone was more recovery than in the dual-infection group. The number of lymphocytes decreased from 14 to 21 dpi with almost no recovery in the dual-infection group. To sum up, *S. suis* contributes to lymphocyte depletion in the ILNs of piglets after HP-PRRSV infection.

HP-PRRSV HuN4 infection induced apoptosis in the ILNs of infected pigs that was enhanced by *S. suis* infection, which may be related to the lymphocyte depletion observed by histopathology. HP-PRRSV is a known immunosuppressive pathogen, reducing the number of lymphocytes in immune organs and in the peripheral blood. It is possible that HP-PRRSV-infected pigs could not clean bacteria in time in the case of lymphatic organs and macrophages damage when *S. suis* invaded, then the bacteria multiply inside the body. *S. suis* is also an immunosuppressive agent (Wang S. et al., 2020), which further destroys the host’s immune organs, resulting in a more severe pathology in immune organs. Thus, our study helps to better understand the pathogenic mechanism of dual-infection commonly seen in the field.

Of the many ways to induce apoptosis (Ghobrial et al., 2005), we focused here on the caspase-3 mediated and the AIF-mediated p53 pathways, and our results revealed apoptosis mediated mainly by caspase-dependent pathways in all the infected piglets. This is the first report to identify which signaling pathway is related to ILN apoptosis in single or dual-infected piglets. Moreover, the expression level of caspase-3 in the dual-infected piglets was significantly higher than in piglets infected with HP-PRRSV alone, indicating a synergy in the pathogenesis caused by *S. suis*. There are reports demonstrating that HP-PRRSV induces apoptosis in bystander cells in the thymus of infected piglets (Li et al., 2014). We found that the HP-PRRSV-infected cells underwent apoptosis, but we did not determine the type of cells (B/T cells or macrophages) that were infected in the ILNs, which warrants further investigation.

Pyroptosis is an important mechanism contributing to pathogens clearance and antimicrobial immunity (Rathinam et al., 2019). Here, we report for the first time that PRRSV HuN4 induces pyroptosis in the ILNs of infected piglets, with fewer pyroptotic cells observed in dual-infected piglets. The levels of proinflammatory cytokines (e.g., IL-1β) in the serum were higher for pigs infected with PRRSV alone compared to pigs coinfected with virus and bacteria (Brockmeier et al., 2017), which is consistent with our results. As we all know, a large number of proinflammatory cytokines are released after pyroptosis. The HP-PRRSV infection group had high levels of proinflammatory cytokines due to the high number of pyroptosis, which warrants verification again in our future coinfection experiments. We theorize that the greater degree of damage to the immune organs observed during dual-infection diminished the host’s ability to clear pathogens and mount an aggressive immune response, and the increased lesions may prevent the cells from mounting a pyroptotic signaling pathway. As a result, the clinical symptoms (cyanotic limbs and ears), gross lesions (interstitial pneumonia, swelling and hemorrhage in ILNs) and microscopic lesions (apoptosis) were more severe in dual-infected piglets than in those infected by HP-PRRSV alone.

## Conclusion

Our results give mechanistic insights into how secondary *S. suis* infection after HP-PRRSV infection can aggravate disease development in affected piglets. We found more caspase-dependent apoptosis in the dual-infected group than in piglets infected with PRRSV alone. The increase in pyroptosis in piglets infected by PRRSV alone shows a clear impact of secondary bacterial infections on pathogen clearance and antimicrobial immunity.

## Data availability statement

The original contributions presented in the study are included in the article/Supplementary material, further inquiries can be directed to the corresponding authors.

## Ethics statement

The animal study was reviewed and approved by the Committee on the Ethics of Animal Experiments of the Harbin Veterinary Research Institute of the Chinese Academy of Agricultural Sciences (CAAS), China.

## Author contributions

SW, MX, TA, and XC conceived the study and designed the experimental procedures. SW, MX, SL, and Y-DT performed the experiments. SW, KY, YZ, and JW analyzed the data. KY, YZ, JW, CL, and XC contributed the reagents and materials. SW, TA, Y-DT, and XC wrote the manuscript. All authors contributed to the article and approved the submitted version.

## Funding

This work was supported by the National Key Research and Development Program of China (2022YFD1800304), National Natural Science Foundation of China (32273018), Central Public-interest Scientific Institution Basal Research Fund (No. 1610302022006), and the Key Program Foundation of Higher Education of Educational Commission of Henan Province (22A230016).

## Conflict of interest

MX was employed by the company Sinopharm Animal Health Corporation Ltd., Wuhan, China.

The remaining authors declare that the research was conducted in the absence of any commercial or financial relationships that could be construed as a potential conflict of interest.

## Publisher’s note

All claims expressed in this article are solely those of the authors and do not necessarily represent those of their affiliated organizations, or those of the publisher, the editors and the reviewers. Any product that may be evaluated in this article, or claim that may be made by its manufacturer, is not guaranteed or endorsed by the publisher.
